# The Most Important Virulence Markers of *Yersinia enterocolitica* and Their Role during Infection

**DOI:** 10.3390/genes9050235

**Published:** 2018-05-03

**Authors:** Agata Bancerz-Kisiel, Marta Pieczywek, Piotr Łada, Wojciech Szweda

**Affiliations:** Department of Epizootiology, Faculty of Veterinary Medicine, University of Warmia and Mazury in Olsztyn, Oczapowskiego 2 Str., 10-719 Olsztyn, Poland; marta.pieczywek@uwm.edu.pl (M.P.); lada_wet@o2.pl (P.Ł.); szweda@uwm.edu.pl (W.S.)

**Keywords:** *Yersinia enterocolitica*, virulence markers, pYV, mucoid *Yersinia* factor MyfA, *Yersinia* adhesin YadA, invasin InvA, attachment-invasion locus protein Ail, *Yersinia* outer membrane proteins Yops, *Yersinia*-stable toxin Yst

## Abstract

*Yersinia enterocolitica* is the causative agent of yersiniosis, a zoonotic disease of growing epidemiological importance with significant consequences for public health. This pathogenic species has been intensively studied for many years. Six biotypes (1A, 1B, 2, 3, 4, 5) and more than 70 serotypes of *Y. enterocolitica* have been identified to date. The biotypes of *Y. enterocolitica* are divided according to their pathogenic properties: the non-pathogenic biotype 1A, weakly pathogenic biotypes 2–5, and the highly pathogenic biotype 1B. Due to the complex pathogenesis of yersiniosis, further research is needed to expand our knowledge of the molecular mechanisms involved in the infection process and the clinical course of the disease. Many factors, both plasmid and chromosomal, significantly influence these processes. The aim of this study was to present the most important virulence markers of *Y. enterocolitica* and their role during infection.

## 1. Introduction

The genus *Yersinia* belonging to the *Enterobacteriaceae* family consists of 18 species, of which only three, *Yersinia pestis*, *Yersinia enterocolitica*, and *Yersinia pseudotuberculosis*, are pathogenic for humans and animals [1]. According to a recent report of the European Food Safety Authority (EFSA), yersiniosis caused by *Y. enterocolitica* is one of the most important foodborne zoonotic diseases in Europe [2]. This pathogenic species has been intensively studied for many years. Six biotypes (1A, 1B, 2, 3, 4, 5) and more than 70 serotypes of *Y. enterocolitica* have been identified to date [3]. The biotypes of *Y. enterocolitica* are divided regarding their pathogenic properties: the non-pathogenic biotype 1A, weakly pathogenic biotypes 2–5, and the highly pathogenic biotype 1B [4]. *Y enterocolitica* infections are influenced by several structures, both plasmid and chromosomal, referred to as virulence markers or virulence determinants [5]. The proteins encoded by these genes enable bacteria to invade a susceptible organism, colonize it, evade the immune response and grow under unfavorable conditions. 

The plasmid of *Yersinia* virulence (pYV) with a size of 64–75 kb is the most known and important virulence marker of *Y. enterocolitica* [1,6]. All biotypes are capable of invading intestinal mucosa, but only strains with a plasmid can migrate from Peyer’s patches to mesenteric lymph nodes and internal organs, where they multiply and lead to the necrotic abscesses formation [7]. Biotype 1B *Y. enterocolitica* strains harboring pYV also carry the chromosomal high pathogenicity island (HPI) associated with the iron acquisition system (yersiniabactin) which facilitates the uptake and utilization of iron by *Y. enterocolitica*, and promotes their growth under iron-limiting conditions [8]. Several genes that are directly responsible for the pathogenicity of *Y. enterocolitica* are located within the pYV, such as *yadA* encoding the *Yersinia* adhesin (YadA), or the *yop* virulon encoding *Yersinia* outer membrane proteins (Yops) [6]. Unfortunately, the determination of the pathogenicity of *Y. enterocolitica* strains based on plasmid markers alone can produce false negative results, due to a spontaneous loss of pYV by bacteria caused by, for example, prolonged strain storage, frequent passaging or temperatures higher than 37 °C [9]. The search for chromosomal, genetically-stable virulence markers, such as *ail*, *invA*, *myfA*, and *yst* genes, which encode the production of Ail (attachment-invasion locus) protein, primary internalization factor invasin InvA, mucoid *Yersiniae* factor MyfA and Yst (*Yersinia*-stable toxin) enterotoxin, respectively, is more justified from the diagnostic point of view [9]. 

Physicochemical parameters, such as temperature, the concentration of calcium and iron ions, pH, and osmolarity also play a role during infection [8,10,11,12]. The expression of many plasmid-encoded virulence genes, selected genes of the flagellar regulon and chromosomal virulence genes, is strictly regulated by temperature [11]. The genes of the flagellar regulon and early virulence genes (*invA*) are expressed below 30 °C, whereas plasmid-encoded virulence genes (*ysc*, *yop*, *yadA*) are expressed at 37 °C [11]. The expression of virulence markers is also regulated by regulatory genes. The most important regulatory gene is *virF*, encoding the transcriptional activator of the *Yersinia* virulence regulon. VirF, a DNA-binding protein of 30 kDa, belonging to the AraC family, is produced only at 37 °C, although other factors are also required to initiate the transcription of VirF target genes [13]. VirF is a key transcriptional activator of the *yop* and *yadA* genes [14]. The *Yersinia* modulator, YmoA, negatively regulates the transcription of *virF* and *invA* [15,16]. Invasion is also mediated by the virulence regulator, RovA, a dimeric winged helix transcriptional regulator which stimulates *invA* expression [17]. Chaperone Hfq modulates the expression of transcriptional regulator *rovA;* therefore, it acts as a global coordinator of surface virulence markers in *Y. enterocolitica*, which suggests that it can be an excellent target for new antimicrobial strategies [17]. The mechanisms of virulence marker expression are generally complicated, and the role of selected regulatory genes and temperature in expression processes is described below.

The aim of this study was to present the most important virulence markers of *Y. enterocolitica* and their role during infection.

## 2. Pathogenesis of *Y. enterocolitica* Infection

Bacteria most often enter the body *per os* with contaminated water or food [1]. In pigs, *Y. enterocolitica* usually colonize palatine tonsils, where they multiply and reach further segments of the gastrointestinal tract. Before the pathogen can come into contact with enteric epithelial cells, bacteria have to penetrate the layer of gastrointestinal mucus which is secreted by goblet cells. Gastrointestinal mucus contains mucins which are responsible for its gel-like properties [18]. Mantle and Husar [19] demonstrated that 1B and 2–5 *Y. enterocolitica* biotypes may adhere to human and rabbit mucin to a greater extent than 1A biotype strains which that do not possess pYV. Lipopolysaccharide (LPS) is encoded chromosomally, it is an integral component of the external cell membrane which forms complex structures with proteins and phospholipids, protects bacterial cells against bile, and complements system components [20]. Free-living *Y. enterocolitica* cells, including cells that enter the intestinal lumen, contain smooth type (S) LPS that varies substantially in the chemical content of the polysaccharide fraction (O-specific chains, O antigen). At 37 °C, LPS is transformed to smooth-rough type (S-R) LPS, which contains less core fraction and O-specific polysaccharide [21,22]. LPS acts as an endotoxin after the breakdown of bacterial cells.

Follicle-associated epithelium (FAE) is the primary site of host–pathogen interactions in *Y. enterocolitica* infection [23]. *Y. enterocolitica* penetrates microfold cells (M cells) and, subsequently, induces the destruction of Peyer’s patches. Autenrieth et al. [23] showed that after *Y. enterocolitica* infection, adjacent villi were dilated from lymphangiectasis, and transmigrating polymorphonuclear leucocytes were found within the epithelium. Interestingly, a pathogenic *Y enterocolitica* serotype 0:8 colonizes ileal Peyer’s patches about 1000 times more densely than surrounding epithelium of a comparable surface area [24]. The FAE and parts of Peyer’s patches are usually destroyed 5–7 days after infection [23]. The target for pathogenic *Y. enterocolitica* are abdominal lymph nodes, where the microorganism multiplies and causes inflammatory changes and ulcerations, and from where it can invade internal organs [23]. 

One of the most intriguing factors, MyfA, plays an important role at the beginning of infection [24]. MyfA closely resembles CS3 fimbriae of enterotoxigenic *Escherichia coli*, which could suggest that MyfA promotes the adhesion to enterocytes [25]. MyfA is also in 44% identical to PsaA, the major subunit of pH6 antigen of *Y. pestis* [25]. The *myfA* gene encoding a fibrillar subunit of MyfA was found in *Y. enterocolitica* strains of bioserotype 4/O:3 isolated from clinical cases of yersiniosis [26]. It was also detected in some *Y. enterocolitica* biotype 1A strains isolated from patients with diarrhea [26]. According to Rastawicki et al. [25], Myf fibrillae are immunogenic at the beginning of disease, and immune responses to recombinant MyfA are more frequent in children than in adult patients.

### 2.1. Adhesion and Invasion

The proteins encoded by three genes, *yadA*, *invA*, and *ail*, are mainly involved in the processes of adhesion and invasion [27]. YadA (previously called YopA) protein is encoded by the structural gene *yadA*, located extrachromosomally on pYV. YadA is a member of the trimeric autotransporter adhesin (TAA) family, and it forms fibrous, lollipop-like structures on the cell surface that mediate binding to human epithelial cells (HEp-2), as well as microvilli that constitute the intestinal brush border [28]. YadA binds collagen I, II, IV, and laminin, and the interactions between YadA and collagen may contribute to chronic *Y. enterocolitica* infections, such as reactive arthritis [5]. YadA supports the creation of densely packed microcolonies of *Yersinia* in three-dimensional collagen gels [29]. Leo et al. [30] examined the binding of YadA to collagen Toolkits, libraries of triple-helical peptides spanning the sequences of type II and III human collagen. YadA was bound to many of these structures, in particular, to hydroxyproline rich peptides [30]. 

YadA also elicits an inflammatory response in epithelial cells by inducing the production of interleukin-8 (IL-8), which is mediated by mitogen-activated protein kinase (MAPK), and by contributing to the intestinal inflammatory cascade [5]. Additionally, YadA mediates cell adhesion and host cell responses induction, like cytokine production, autoagglutination, and serum resistance [30,31,32,33]. Mühlenkamp et al. [33] demonstrated direct YadA-mediated interactions between selected *Y. enterocolitica* strains with serum glycoprotein vitronectin (Vn), which acts as an inhibitory regulator of the terminal complement complex. According to these authors, YadA-mediated Vn binding is caused by the “uptake region” of the protein’s N-terminus, and it promotes the adhesion of selected *Y. enterocolitica* strains. This “uptake region”, similar to the “uptake region” of *Y. pseudotuberculosis* YadA, was defined as a crucial for the high-affinity binding of Vn [33]. It is worth mentioning that YadA plasmid protein is produced at 37 °C.

The production of YadA is promoted by the RNA chaperone Hfq, a widely conserved protein that stabilizes sRNAs, facilitates sRNA–mRNA pairing, and modulates the degradation of target RNAs [34]. According to Kakoschke et al. [17], Hfq is probably the main regulator in *Yersinia*. They observed that *hfq* mutants exhibited decreased translocation of proteins into host cells by the type III secretion system (T3SS), consistent with decreased YadA production [17]. They assumed that Hfq-dependent control exerted at the transcriptional (at 27 °C), post-transcriptional (at 37 °C in stationary phase) and, possibly, post-translational (37 °C in stationary phase) level is involved in fine-tuning the amount of YadA present at the bacterial surface in different environments. Nieckarz et al. [35] demonstrated that another regulatory system, OmpR, can specifically bind to the *yadA* promoter region, which suggests that expression is inhibited by a direct mechanism. They identified *yadA* as a new member of the OmpR regulon, and postulated that OmpR negatively regulates *yadA*, and that the downregulation of YadA could increase the survival of *Y. enterocolitica* by preventing bacterial binding to host cells, which promotes further spread to deeper tissues. To summarize, YadA seems to be the most important single contributor to the virulence of *Y. enterocolitica*, in particular, because it acts not only as an adhesin, but also as an invasin to facilitate the penetration of host cells [28]. YadA also plays a central role in promoting serum resistance. Kirjavainen et al. [36] showed that YadA acted as C4-binding protein (C4bp) receptors, and binding of C4bp could help *Y. enterocolitica* to evade complement-mediated clearance in the human host. Kinetic killing tests, which were conducted by Biedzka-Sarek et al. [37], revealed that the most potent single-serum resistance factor needed for long-term survival was YadA.

Some studies showed that in an early phase of *Y. enterocolitica* infection, InvA is required for effective bacteria translocation into M cells and Peyer’s patches colonization [38]. The 987 aminoacids polypeptide InvA, a member of the intimin/invasin protein family, consists of a membrane-associated β-barrel and extracellular C-terminal domains, and its production is encoded by the *invA* chromosomal gene [39]. The β-barrel at the N-terminus and the extracellular domain at the C-terminus invert the arrangement of InvA relative to the classical autotransporter system (type Va secretion system), which could represent a new type of autotransporter system (Ve) [40]. InvA binds to integrins, which leads to the creation of integrin clusters, triggers remodeling of the actin cytoskeleton and leads to the internalization of *Y. enterocolitica* to epithelial cells. The above is known as the “zipper” invasion mechanism, and internalization allows the delivery of Yops to host cells [41].

The penetration of M cells by *Y. enterocolitica* begins with a direct interaction between InvA and any of the five types of β_1_ integrins (α_3_β_1_, α_4_β_1_, α_5_β_1_, α_6_β_1_, α_v_β_1_) on the surface of target epithelial cells [42]. InvA binds directly, whereas YadA binds indirectly via extracellular matrix (ECM) proteins to β_1_ integrins on host cells [43]. According to Thinwa et al. [44], the invasin–integrin interaction provides the first signal for inflammasome activation, and the T3SS translocon provides the second signal for inflammasome activation, which results in the release of IL-18. The binding of InvA to β_1_ integrins rapidly induces IL-18 mRNA expression, which could suggest that integrins provide the first signal for inflammasome activation [44]. After binding, InvA activates multiple signaling cascades, which leads to the chemotactic cytokines production like IL-8, monocyte-chemoattractant protein-1 (MCP-1) and the granulocyte-macrophage colony-stimulating factor [45]. According to Thinwa et al. [44], the inflammatory reaction triggered by *Yersinia* InvA leads to the recruitment of phagocytes and tissue disruption. Wiedemann et al. [46] demonstrated that after coming into contact with β_1_ integrins, InvA induces phagocytosis of nonopsonized *Y. enterocolitica* by activating actin polymerization. *Y. enterocolitica* resists macrophage killing, becomes sequestered inside these cells, and the invasin-triggered recruitment of macrophages creates replicative niches for *Y. enterocolitica* and enables the pathogen to avoid exposure to the host’s immune system [47]. According to Deuretzbacher et al. [48], InvA also mediates the induction of autophagy in macrophages.

InvA synthesis in *Y. enterocolitica* is dependent on the growth phase and, according to recent research, on serotype. Uliczka et al. [27] observed that in serotypes O:8 and O:9, InvA was expressed efficiently only at environmental temperatures, whereas in serotype O:3, InvA production was constitutive also at higher temperatures. These authors found that the difference in regulation resulted from the insertion of IS1667 into the *invA* promoter region in *Y. enterocolitica* serotype O:3. However, Pepe et al. [10] demonstrated that pH was also an important determinant of *invA* expression. *invA* is expressed maximally at 26 °C and neutral pH, and is only poorly expressed at 37 °C and neutral pH; however, a decrease in pH increases *invA* expression at 37 °C. The expression of InvA in response to environmental signals is regulated by several proteins, and genetic studies have revealed the complex nature of this regulatory process [49]. *InvA* expression is regulated mostly by RovA. Single substitution of amino acid P98S increases the thermostability of RovA, leading to higher expression of InvA in serotype O:3 [27]. Admittedly, Raczkowska et al. [50] demonstrated that RovA may activate *invA* expression regardless of the presence of histone-like nucleoid structuring (H-NS) protein, however, in general, the thermoregulation of *invA* gene transcription in *Y. enterocolitica* is controlled by three regulatory proteins: RovA, H-NS, and YmoA [51]. RovA also upregulates *yaxA* and *yaxB* genes [52]. Wagner et al. [52] found that YaxA and YaxB proteins are required for cytotoxic activity, and that they are associated. YaxAB acts as a virulence factor by inducing cell lysis through the creation of pores in the host cell membrane [52].

Ail protein is yet another virulence marker which plays an important role during attachment and invasion processes. Due to its small size (17 kDa), Ail protein is easily masked by other surface structures, like the LPS, decreasing its biological efficiency [37]. Ail has eight membrane-spanning amphipathic β-strands and four extracellular loops. The C-terminal half of loop 2 is involved in interactions with host cell surface components [47]. Miller et al. [53] showed that mutations in loop 2 of Ail lead to the elimination of the invasion phenotype and serum resistance of *Y. enterocolitica*. During the logarithmic phase of growth, Ail is synthesized at 30 °C; although in the stationary phase, the *ail* gene is only expressed at 37 °C [41]. Ail mediates the attachment and invasion of host cells and confers serum resistance to *Y. enterocolitica* [54]. Ail in combination with YadA guarantees a high level of serum resistance by binding factor H, an abundant serum glycoprotein essential for controlling the alternative pathway in blood and on cell surfaces [52,55]. Ail is also the key determinant of serum resistance during exponential growth [52]. It confers serum resistance to *Y. enterocolitica* by binding C4bp, a complement component, which inactivates C3 convertase and destabilizes the formation of active attack complexes on the bacterial membrane [55]. Bliska and Falkow [56] examined the *ail* mutant of *Y. enterocolitica* and the expression of the *ail* gene in *E. coli*, and observed that Ail increased serum resistance 100-fold. O-group LPS side chains sugars could mask the Ail receptor-binding regions, thereby weakening its serum resisting properties, but the loss of O-group sugars at 37 °C exposes Ail [47]. Because of these temperature-dependent changes, the cells cultured at 37 °C are more resistant to serum killing than the cells cultured at 30 °C [57]. In contrast to YadA and InvA, chaperone Hfq inhibited the production of Ail post-transcriptionally [17].

The *ail* gene is an important virulence marker which is widely used as a target in molecular analyses of the pathogenicity of *Y. enterocolitica* strains [58,59,60]. In contrast to the *invA* sequence, sequences homologous to the *ail* gene have been identified only in *Y. enterocolitica* strains that are epidemiologically associated with clinical yersiniosis in humans, however, they are now more often detected in the 1A biotype [3,61,62,63,64]. According to Sihvonen et al. [63], the nucleotide sequence of the *ail* gene of a biotype 1A *Y*. *enterocolitica* strain (isolated from food in Finland, GenBank FN812733) differed from a biotype 1B *Y*. *enterocolitica* strain (isolated from a human with clinical yersiniosis, GenBank AM286415) in only one point mutation—transition G2008088T. This finding suggests that the pathogenicity of *Y. enterocolitica* cannot be reliably determined based on *ail* detection in simple PCR. Bancerz-Kisiel et al. [4] recently proposed the high-resolution melting analysis (HRMA) method supporting rapid determination of *ail* single nucleotide polymorphisms (SNPs) correlated with the biotypes of the tested *Y. enterocolitica* strains. According to these authors, the HRMA supported reliable discrimination of three genotypes and phylogenetic groups: 1A—non-pathogenic *Y. enterocolitica* strains, 1B—highly pathogenic *Y. enterocolitica* strains, and 2/4—weakly pathogenic *Y. enterocolitica* strains [4]. This method could pose an alternative to standard biotyping, serotyping, and sequencing of *ail*-positive *Y. enterocolitica* strains.

### 2.2. Interaction with the Immune Response

Antigen transcytosis across the FAE of Peyer’s patches by M cells is significant for invasion and induction of effective immune responses to mucosal antigens [65]. Bacteria transported by M cells to Peyer’s patches are surrounded, ingested, and destroyed by phagocytic cells. Antigens are presented to lymphocytes by antigen presenting cells (APC) in Peyer’s patches, which leads to the induction of the immune response and, ultimately, local and systemic mucosal immunity. However, enteropathogenic *Y. enterocolitica* strains have developed resistance mechanisms, mainly with the involvement of effector Yops encoded by the *yop* virulon. The *yop* virulon has four components: a type III secretion system, T3SS, known as Ysc-Yops, translocator Yops (YopB, YopD), control element YopN, and effector Yops (YopE, YopH, YopM, YopO, YopP, YopT) [66]. Selected Yops form pores in the membrane of eukaryotic target cells, whereas other Yops are effector proteins that are transported through the pores to the cytosol of target cells [67]. The transport of Yops also requires close contact between bacterial and host cells, and it is mediated by YadA and InvA that bind to β_1_ integrins [5].

The T3SS transports Yops to the cytoplasm of host cells through a syringe-like system, formed mainly by YscF and translocator Yops [66,68]. YopB and YopD, two similar hydrophobic proteins referred to as translocators, form a multimeric integral membrane complex in the membrane of eukaryotic cells [69]. YopB acts as a potential suppressor of tumor necrosis factor alpha (TNF-α) mRNA expression in macrophages and Peyer’s patches in vivo, and it has also been found to play a role in the regulation and/or secretion of YopD [70]. *Y. enterocolitica* strains lacking YopD are unable to induce cytotoxicity in macrophages. These properties were not inherent to YopD, but indirectly related to its role in the secretion of YopE and YopH into host cells [70]. YopN, a secreted protein of 32.3 kDa, is a multi-domain protein with several functions, including the control of Yops secretion [71]. Effector Yops have numerous properties which enable them to block local immune mechanisms. After transport, they inhibit phagocytosis and the production of inflammatory molecules, and they induce host cell apoptosis. YopE, YopH, YopO and YopT have a negative role in cytoskeleton dynamics [67]. YopE has been notified to regulate the translocation of effector Yops into host cells [72]. YopH promotes the inhibition of phagocytosis, inhibits cytokine production by T cells and T-cell proliferation, and prevents the expression of the costimulatory receptor CD86 on B cells [73]. YopO inhibits phagocytosis by disrupting actin filament regulation processes [74], and YopT disrupts the actin cytoskeleton of the host cell [75]. YopM is a crucial immunosuppressive effector of pathogenic *Yersinia*: it enters the nucleus of host cells, but the mechanisms governing its nucleocytoplasmic shuttling and its intranuclear activities have not been explained to date [76]. YopP has numerous roles: it suppresses the production of TNF-α and IL-8, blocks the activation of MAPK and nuclear factor κB (NF-κB), and induces apoptosis in macrophages [67].

The injection of Yops into host cells by T3SS is an important immune evasion mechanism of *Y. enterocolitica*. Although leukocytes are the target of Yops injected by *Y. enterocolitica*, it remains unknown which adhesins and leukocyte receptors are required for Yops injection. Deuschle et al. [43] investigated the role of YadA, InvA, and β_1_ integrins in Yops injection into leukocytes. Based on the results of a β-lactamase reporter assay, they found that the adhesion of *Y. enterocolitica through* InvA or YadA is sufficient to promote Yops injection into leukocytes. Serum factors inhibit YadA-mediated, but not InvA-mediated Yops injection into B and T cells, and YadA-mediated Yops injection is shifted towards neutrophils and other myeloid cells [43]. The above authors also found that YadA is essential for *Y. enterocolitica* virulence and Yops injection into leukocytes, whereas InvA is not required for virulence, and plays only a temporary and minor role in the injection of Yops in the early phase of infection [43]. Deuschle et al. [43] demonstrated that β_1_ integrins are not required for YadA-mediated Yops injection into leukocytes, but they contribute to InvA-mediated Yops injection. Keller et al. [77] reported that in the absence of β_1_ integrins, YadA mediates Yops injection by interacting with αV integrins, and unidentified cofactors expressed by epithelial cells, but not fibroblasts. They revealed that by indirectly binding to a broad range of ECM host cell receptors in different cell types, YadA is a versatile tool for Yops injection [77].

### 2.3. Diarrhea Induction

Enterotoxin Yst is one of the significant factors determining *Y. enterocolitica* virulence. Yst is a polypeptide chain composed of a 30 amino acid C-terminal domain as the mature component of the toxin and an 18 amino acid N-terminal signal sequence that is cut off during transport through the cytoplasmic membrane [78]. Three types of YstI enterotoxins (A, B, and C) and a recently discovered, but weakly understood YstII enterotoxin, have been identified to date [79]. *Y. enterocolitica* strains of biotypes 1B and 2–5 produce the YstIA heat-stable enterotoxin [80,81]. Research into YstIA-negative mutants and their transcription demonstrated that YstIA could be essential for virulence in *Y. enterocolitica* [82,83]. Biotype 1A strains produce mainly YstIB, and they rarely produce YstIC, which is not commonly encountered [84]. YstII, a biologically active enterotoxin with a completely different mechanism of action and an unidentified coding gene, has also been identified [80]. YstIC consists of 53 amino acids, has higher molecular mass, and C-terminal and N-terminal YstIC and YstIA chains share approximately 50% similarity at the amino acid level. The C-terminal 13 amino acid regions of YstIA and YstIB correspond to a strongly conservated sequence that is characteristic of all thermostable toxins produced by enterotoxigenic *E. coli* [85].

Pathogenic *Y. enterocolitica* strains isolated from humans with yersiniosis were capable of producing YstI, which implies that YstI plays a significant role in the etiology of diarrhea in yersiniosis. Researchers opposing the theory that YstI is the key determinant of diarrhea have pointed out that YstI is not produced at temperatures higher than 30 °C. However, Singh et al. [81] demonstrated that YstI can be produced both at 37 °C in a slightly alkaline environment (pH 7.5) and at temperatures below 30 °C. The YstIA enterotoxin has highly similar physicochemical and antigenic properties and mechanism of action to the heat-stable enterotoxin type I or type a (STI, STa) produced by enterotoxigenic *E. coli*, which are manifested by the activation of guanylate cyclase, an increase in cyclic guanosine monophosphate (cGMP) levels in epithelial cells, and the accumulation of fluids in the intestinal lumen, which leads to diarrhea [78,86]. Interestingly, not all *yst-*positive strains produce enterotoxins, which could imply the presence of “silent genes”. This can probably be attributed to the presence of the *ymoA* gene, which encodes the production of YmoA protein (8.1 kDa). The YmoA protein belongs to the growing family of Hha proteins, referred to as YmoA in *Y. enterocolitica*, and it is similar to the N-terminal, dimerized domains of H-NS proteins [87,88]. In intestinal bacteria, H-NS proteins play a host of important roles as structural proteins and gene expression modulators, including those of virulence markers [89,90,91,92,93,94]. There is evidence to suggest that *ymoA* inhibits the expression of the *invA* gene, and participates in VirF regulation [16] and temperature-dependent production of Yops and YadA [15].

The possible effect of *ymoA* gene mutations on YstIA production was first postulated by Cornelis et al. [15]. They identified two Tn5-Tc1 chromosomal mutants responsible for producing YstI with its typical temperature dependence. These mutations were insertions in *ymoA* gene. Cornelis et al. [15] postulated that *ymoA* mutations unblock the silencing of the *yst* gene and stimulate YstI production. Mikulskis et al. [83] also postulated the inherence of a mechanism switching the *yst* expression to a silent state, and other authors showed that *yst* gene presence is not always correlated with YstI production [85,95]. In the study of Bancerz-Kisiel et al. [96], no mutations were found in the coding region of the examined *ymoA* gene fragments. Two point mutations in the non-coding region have been identified in some of the tested *Y. enterocolitica* strains, regardless of their enterotoxic properties [96]. Hence, the suggestion that *ymoA* gene mutations influence *ystA* gene silencing was not confirmed.

### 2.4. Pathogenicity of Y. enterocolitica Biotype 1A Strains

*Y. enterocolitica* strains belonging to biotype 1A are commonly regarded as non-pathogenic; they are highly heterogeneous and include many O serogroups [95]. Biotype 1A strains isolated from clinical cases also colonize the gastrointestinal tract, both small and large intestines, and replicate in enterocytes. Despite the fact that *Y. enterocolitica* strains belonging to biotype 1A are regarded as avirulent and devoid of pYV, various forms of fimbriae have been observed in this biotype [8]. Biotype 1A strains isolated from clinical cases of gastritis and enteritis show higher virulence and capacity to invade enterocytes than environmental strains [95]. They penetrate HEp-2 cells less effectively than the strains that possess the pYV plasmid, but more effectively than strains obtained from other sources [80]. This suggests a different mechanism of epithelial cell invasion than in the biotypes that are regarded as pathogenic [81]. The pathogens escape from macrophages or epithelial cells without causing detectable cytolysis, which implies the presence of an escape mechanism resembling exocytosis [97,98]. The fact that clinical biotype 1A *Y. enterocolitica* strains are much more resistant to macrophage killing and more likely to escape from host cells than nonclinical strains suggest that these properties may be responsible for virulence [97,98]. Although the 1A biotype rarely produces YstIA enterotoxin, more than 80% of these strains contain the *ystB* gene that encodes the production of homologous and biologically active enterotoxin YstIB. Rammamurthy et al. [99] showed that 88.9% of clinical *Y. enterocolitica* strains belonging to biotype 1A caused the accumulation of fluids in the intestines of suckling mice, which points to the toxigenic potential of the tested strains. Additionally, the minimum effective dose (MED) of purified YstIB enterotoxin (0.4 pmol) is much lower than an analogous dose of YstIA enterotoxin (7.6 pmol), which confirms its pathogenic potential [59]. According to McNally et al. [100], biotype 1A strains are emerging as the predominant pathogenic agent of yersiniosis in the Commonwealth countries. A case-control study of diarrheic patients in Finland also revealed that the majority of the isolated *Y. enterocolitica* strains belonged to biotype 1A [101].

The above suggests that pathogenicity cannot be reliably determined based on bioserotype classification alone.

**Figure 1:**
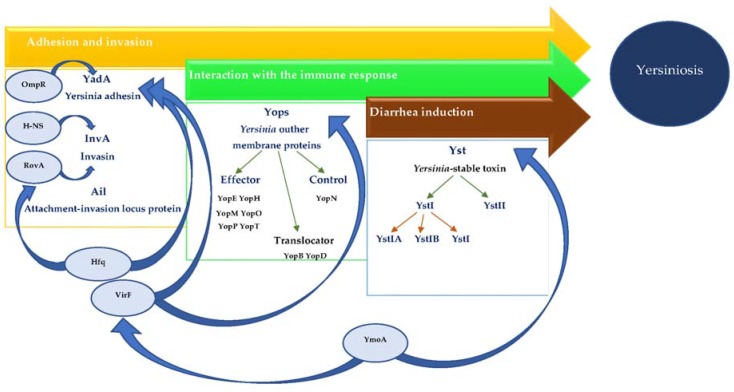
The role of selected regulatory genes in *Yersinia enterocolitica* virulence markers expression.

Major virulence markers of *Yersinia enterocolitica* are summarized in Table 1, and the role of selected regulatory genes in their expression is presented on Figure 1.

## 3. Conclusions

*Yersinia enterocolitica* is the causative agent of yersiniosis, a zoonotic disease of growing epidemiological importance with significant consequences for public health. Due to its complex pathogenesis, further research is needed to expand our knowledge of the molecular mechanisms involved in the infection process and the clinical course of the disease. Many factors, both plasmid and chromosomal, significantly influence these processes.

## Figures and Tables

**Table 1:** Major virulence markers of *Y. enterocolitica.*

Gene	Gene Product	Plasmid/Chromosomal
*myfA*	mucoid *Yersiniae* factor (MyfA)	Chromosomal
*yadA*	*Yersinia* adhesin (YadA)	Plasmid
*invA*	invasin (InvA)	Chromosomal
*ail*	attachment-invasion locus (Ail) protein	Chromosomal
*yop* virulon	*Yersinia* outer membrane proteins (Yops)	Plasmid
*ystA*	*Yersinia*-stable toxin type I A (YstIA)	Chromosomal
*ystB*	*Yersinia*-stable toxin type I B (YstIB)	Chromosomal
*ystC*	*Yersinia*-stable toxin type I C (YstIC)	Chromosomal
*virF*	transcriptional activator of the *Yersinia* virulence regulon (VirF)	Plasmid
*ymoA*	*Yersinia* modulator (YmoA)	Chromosomal
